# Rectus Sheath Hematoma as a Cutaneous Manifestation of Essential Thrombocythemia

**DOI:** 10.7759/cureus.81324

**Published:** 2025-03-27

**Authors:** Shin Iinuma, Takahiro Kobayashi, Takahiro Nagashima

**Affiliations:** 1 Dermatology, Japanese Red Cross Kitami Hospital, Kitami, JPN; 2 Dermatology, Asahikawa Medical University, Asahikawa, JPN; 3 Internal Medicine, Japanese Red Cross Kitami Hospital, Kitami, JPN

**Keywords:** essential thrombocythemia, hemorrhagic complications, myeloproliferative neoplasm, rectus sheath hematoma, thrombocytosis

## Abstract

Rectus sheath hematoma (RSH) is a rare cause of acute abdominal pain that is often associated with trauma, anticoagulant therapy, or coagulopathy. We present a unique case of an RSH as a cutaneous manifestation of essential thrombocythemia (ET), which is a myeloproliferative neoplasm characterized by thrombotic and hemorrhagic complications. A 66-year-old female patient presented with acute lower abdominal pain, a subcutaneous mass, and overlying skin ecchymosis. Imaging confirmed a significant RSH. Laboratory tests revealed severe anemia and marked thrombocytosis. Further evaluations, including bone marrow biopsy and genetic testing, revealed ET with a *CALR* mutation. RSH was managed conservatively, and cytoreductive therapy comprising hydroxyurea was administered for ET, resulting in resolution of the hematoma and improvement in hematological parameters. This case highlights the necessity of considering hematological disorders when patients present with spontaneous hematomas. Prompt diagnosis and effective management of both the hematoma and ET were achieved, demonstrating that timely recognition and appropriate treatment are important to improving patient outcomes.

## Introduction

Rectus sheath hematoma (RSH) is a rare condition caused by bleeding into the rectus sheath, which is a fibrous compartment that encloses the rectus abdominis muscles along the anterior abdominal wall. RSH often mimics an acute abdomen, presents as a palpable abdominal mass, and poses the potential risk of life-threatening complications. Common etiological factors include trauma, abdominal surgery, anticoagulant or antiplatelet therapy, and, less frequently, underlying hematological disorders [1]. Essential thrombocythemia (ET) is an acquired myeloproliferative neoplasm characterized by clonal thrombocytosis and an increased risk of thrombotic and hemorrhagic complications. The *CALR* mutation is found in a subset of ET cases and contributes to the disease pathogenesis by promoting abnormal megakaryocyte proliferation and altered cytokine signaling [2]. This report describes a rare presentation of an RSH as a cutaneous manifestation of ET, thus emphasizing its role as an observable clinical feature rather than an incidental finding. Furthermore, this case highlights the importance of considering hematological disorders in the differential diagnosis of spontaneous hematomas and emphasizes the critical role of timely recognition and appropriate management in improving outcomes of complex cases.

## Case presentation

A 66-year-old female patient presented to our Dermatology Department with severe lower abdominal pain and a subcutaneous mass. The pain had started abruptly upon waking on the day of admission. The patient’s medical history included pulmonary carcinoma that had been surgically treated one decade earlier and was in remission without ongoing treatment, as well as a six-month history of chronic urticaria managed with oral olopatadine (5 mg twice daily) and bilastine (20 mg daily). The patient denied a history of abdominal surgery and the use of anticoagulant or antiplatelet medications.

**Figure 1:**
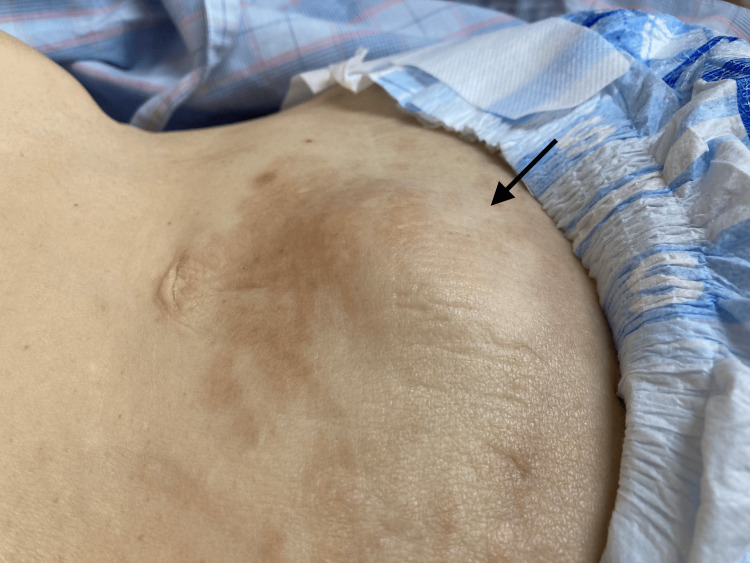
Clinical presentation Physical examination reveals a firm, palpable mass with a measurement of approximately 8 cm × 5 cm in the subumbilical region (arrow). Mild ecchymosis is observed on the overlying skin.

The patient’s vital signs were within normal limits. A physical examination revealed significant tenderness, rebound tenderness, and a firm, palpable, approximately 8 cm × 5 cm mass in the subumbilical region (Figure 1). Mild ecchymosis was observed on the overlying skin.

**Figure 2:**
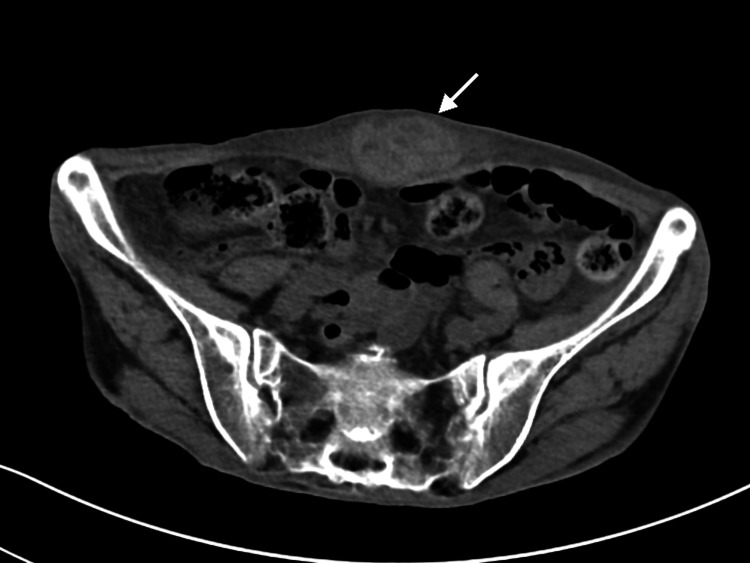
Computed tomography image Abdominal and pelvic computed tomography image shows an 8 cm × 5 cm × 3.5 cm heterogeneous area with relatively high attenuation within the rectus sheath musculature (arrow) consistent with a rectus sheath hematoma. The normal rectus muscle appears uniform and does not have such collections.

Abdominal and pelvic computed tomography identified an 8 cm × 5 cm × 3.5 cm heterogeneous area with relatively high attenuation within the rectus sheath musculature, consistent with a diagnosis of RSH (Figure 2).

**Figure 3:**
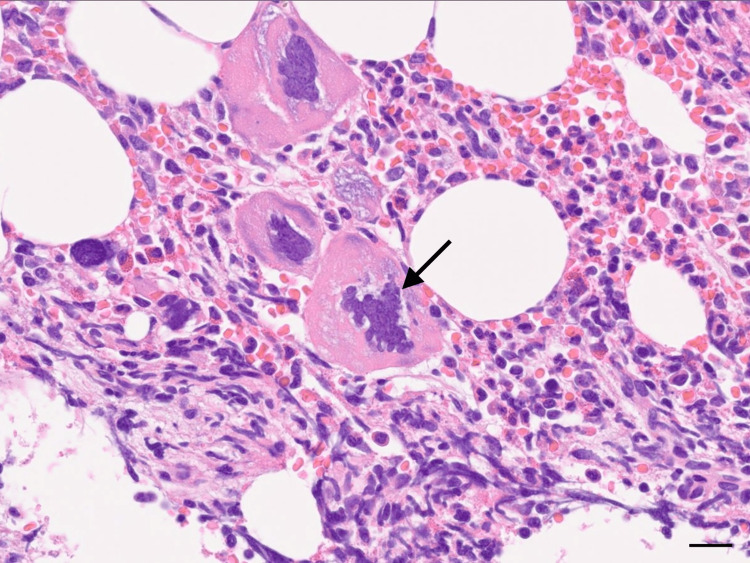
Bone marrow biopsy results A bone marrow biopsy revealed an increased number of enlarged mature megakaryocytes with hyperlobulated nuclei (arrow) (hematoxylin and eosin stain; original magnification ×400; scale bar: 20 µm). These findings are typical of essential thrombocythemia and differ from those of normal marrow, which has fewer and smaller megakaryocytes.

Laboratory tests revealed a hemoglobin level of 6.8 g/dL (reference range: 11.4-14.8 g/dL); therefore, transfusion comprising 4 units of packed red blood cells was required. A markedly increased platelet count of 1,547,000/μL (reference range: 158,000-348,000/μL) was also observed. The patient was referred to a hematologist for further evaluation. A bone marrow biopsy revealed an increased number of enlarged mature megakaryocytes with hyperlobulated nuclei (Figure 3).

Laboratory tests revealed a von Willebrand factor antigen level of 96% (reference range: 55%-155%), von Willebrand factor activity level of 66% (reference range: 60%-170%), and factor VIII activity level of 240% (reference range: 70%-140%), which do not support a diagnosis of acquired von Willebrand syndrome. Genetic studies identified a *CALR* mutation (type I), but the results were negative for *JAK2* and *MPL* mutations. These findings confirmed the diagnosis of ET. Cytoreductive therapy comprising hydroxyurea was initiated and resulted in improvements in the hematological abnormalities.

The patient’s abdominal symptoms resolved significantly over the course of three weeks, and follow-up imaging demonstrated substantial contraction of the hematoma. Careful observation during one year of follow-up revealed no bleeding recurrence or other hematological complications.

## Discussion

RSH is an uncommon condition caused by bleeding into the rectus sheath that is typically caused by the rupture of the superior or inferior epigastric arteries. RSH occurs when these vessels or their perforating branches are compromised because of trauma, iatrogenic injury, or spontaneous causes. The primary clinical manifestations of RSH include acute abdominal pain and a palpable mass that is often located in the lower abdomen. Patients may present with anemia or hemorrhagic shock if the hematoma expands significantly. The tamponade effect provided by the rectus sheath usually limits the extent of bleeding; however, in some cases, RSH can be life-threatening. Physical examination frequently reveals a tender, firm mass in the abdominal wall, whereas ecchymosis is infrequently observed [3]. Notably, our patient initially presented to our Dermatology Department because of a subcutaneous mass and pain, thus highlighting the diagnostic challenge associated with RSH and the importance of considering RSH when evaluating abdominal wall abnormalities in atypical clinical settings. Computed tomography is the gold standard for diagnosing RSH because it allows precise evaluation of the size, extent, and location of the hematoma and excludes other differential diagnoses such as appendicitis, diverticulitis, pancreatitis, pelvic inflammatory disease, and neoplastic processes. In certain instances, the hematoma may cross the midline and present bilaterally, as observed in the current case [4].

The development of RSH is associated with multiple risk factors, including anticoagulant or antiplatelet therapy, advanced age, female sex, abdominal trauma or surgery, cancer, and coagulopathies [5]. In the present case, ET was identified as the underlying cause of RSH, thus representing an exceedingly rare presentation. ET, which is a myeloproliferative neoplasm characterized by excessive proliferation of megakaryocytes, leads to thrombocytosis. Clinical manifestations of ET include thrombotic complications (e.g., cerebrovascular events, myocardial infarction) and hemorrhagic complications (e.g., gastrointestinal bleeding). Although less common, spontaneous soft tissue or muscular hematomas have been reported as bleeding manifestations with ET [6]. Hemorrhagic events that occur with ET are occasionally associated with acquired von Willebrand syndrome, which was excluded as a diagnosis for this patient [7,8]. Therefore, in this case, several potential mechanisms of platelet dysfunction were considered contributing factors, including acquired storage pool deficiency, increased platelet activation, decreased adrenergic receptor expression, impaired response to epinephrine, and reduced expression of platelet membrane glycoprotein receptors [2]. The diagnosis of ET requires a comprehensive evaluation, including peripheral blood smear analysis, bone marrow biopsy, and genetic testing for mutations of *JAK2*, *CALR*, or *MPL*. Cytoreductive therapy or treatment comprising antiplatelet agents, depending on the risk stratification, is administered to reduce the thrombotic and hemorrhagic risks [9]. In the present case, hydroxyurea was administered to manage the underlying ET and prevent future complications. Other treatment options for ET include interferon-alpha and busulfan, depending on the age and clinical status of the patient.

In addition to hemorrhagic and thrombotic manifestations, ET and other myeloproliferative neoplasms cause pruritus, which may be triggered by abnormal cytokine production and increased histamine release. In this case, the patient had chronic urticaria managed with antihistamines, but it is possible that her pruritic symptoms were partially related to ET rather than being an entirely separate dermatologic condition. Because the exact relationship remains uncertain, further investigations of ET-associated pruritus and its underlying mechanisms are necessary.

The management of RSH depends on the patient’s hemodynamic stability [10]. Patients who are hemodynamically stable are typically treated conservatively with bed rest, analgesia, fluid resuscitation, and blood transfusions as needed, as well as correction of any underlying coagulopathy. CT imaging plays a crucial role in guiding management decisions by assessing the hematoma size and extent, and active extravasation. Angiographic embolization of the bleeding vessel is often the first-line intervention for cases involving hemodynamic instability or evidence of active bleeding (e.g., contrast extravasation on CT). Surgical evacuation is considered for rare refractory cases or when embolization fails [11]. Conservative management and treatment of the underlying ET were sufficient for our patient; therefore, invasive intervention was not required.

## Conclusions

This case illustrates the diagnostic complexity of RSH with cutaneous manifestations that ultimately indicated the diagnosis of ET. The association between severe thrombocytosis and acquired coagulopathy in ET highlights the importance of considering hematological disorders when patients present with spontaneous hematomas. Imaging and a hematological evaluation facilitated the prompt diagnosis and effective management of both the hematoma and ET. Conservative treatment successfully resolved the RSH, whereas cytoreductive therapy addressed the systemic condition. This case highlights the need to evaluate the systemic causes of atypical clinical presentations.
